# Supplementary material for: Optimization of extraction conditions and determination of purine content in marine fish during boiling
Source: PeerJ. 2019 May 6;7:e6690. doi: 10.7717/peerj.6690 (PMC6507899; doi:10.7717/peerj.6690)
Supplement: Supplemental Information 2 — aTotal extraction yield (%) = Adenine extraction yield + Guanine extraction yield + Hypoxanthine extraction yield + Xanthine extraction yield bExtraction yield (%) = (purine content in sample (mg)/sample mass (kg)) × 100 [file peerj-07-6690-s002.docx]

Table S1. The Box–Behnken experimental design of PCA method with four independent variables

| No. | PCA concentration (%) | Hydrolysis temperature (℃) | Hydrolysis time（min） | Liquid-solid ratio (ml/g) | ^a^Total extraction yield |
| --- | --- | --- | --- | --- | --- |
| 1 | 1 (90) | 1 (90) | 0 (55) | 0 (60) | 83.11 |
| 2 | -1 (70) | 0 (80) | 1 (60) | 0 (60) | 82.00 |
| 3 | -1 (70) | 1 (90) | 0 (55) | 0 (60) | 82.13 |
| 4 | 0 (80) | 0 (80) | 0 (55) | 0 (60) | 85.97 |
| 5 | -1 (70) | 0 (80) | -1 (50) | 0 (60) | 80.43 |
| 6 | 0 (80) | 0 (80) | 1 (60) | 1 (70) | 85.85 |
| 7 | -1 (70) | 0 (80) | 0 (55) | -1 (50) | 83.83 |
| 8 | 0 (80) | 0 (80) | 0 (55) | 0 (60) | 86.12 |
| 9 | 0 (80) | 0 (80) | -1 (50) | -1 (50) | 85.13 |
| 10 | 0 (80) | 0 (80) | 1 (60) | -1 (50) | 85.31 |
| 11 | 0 (80) | 0 (80) | -1 (50) | 1 (70) | 83.99 |
| 12 | 0 (80) | 1 (90) | 1 (60) | 0 (60) | 84.12 |
| 13 | 0 (80) | 1 (90) | -1 (50) | 0 (60) | 84.03 |
| 14 | 1 (90) | 0 (80) | 0 (55) | 1 (70) | 83.03 |
| 15 | 0 (80) | -1 (70) | 0 (55) | 1 (70) | 84.42 |
| 16 | 0 (80) | 0 (80) | 0 (55) | 0 (60) | 85.92 |
| 17 | 0 (80) | 1 (90) | 0 (55) | 1 (70) | 84.83 |
| 18 | 1 (90) | 0 (80) | 0 (55) | -1 (50) | 83.32 |
| 19 | 1 (90) | 0 (80) | -1 (50) | 0 (60) | 82.95 |
| 20 | -1 (70) | -1 (70) | 0 (55) | 0 (60) | 82.11 |
| 21 | 0 (80) | 0 (80) | 0 (55) | 0 (60) | 85.21 |
| 22 | 1 (90) | -1 (70) | 0 (55) | 0 (60) | 83.43 |
| 23 | 0 (80) | -1 (70) | 1 (60) | 0 (60) | 84.15 |
| 24 | 1 (90) | 0 (80) | 1 (60) | 0 (60) | 82.43 |
| 25 | 0 (80) | -1 (70) | 0 (55) | -1 (50) | 85.00 |
| 26 | 0 (80) | -1 (70) | -1 (50) | 0 (60) | 84.12 |
| 27 | -1 (70) | 0 (80) | 0 (55) | 1 (70) | 82.12 |
| 28 | 0 (80) | 0 (80) | 0 (55) | 0 (60) | 86.01 |
| 29 | 0 (80) | 1 (90) | 0 (55) | -1 (50) | 85.77 |

^a^Total extraction yield (%) = Adenine extraction yield + Guanine extraction yield + Hypoxanthine extraction yield + Xanthine extraction yield

^b^Extraction yield (%) = (purine content in sample (mg)/sample mass (kg)) × 100
