# Supplementary material for: Optimization of extraction conditions and determination of purine content in marine fish during boiling
Source: PeerJ. 2019 May 6;7:e6690. doi: 10.7717/peerj.6690 (PMC6507899; doi:10.7717/peerj.6690)
Supplement: Supplemental Information 3 — aTotal extraction yield (%) = Adenine extraction yield + Guanine extraction yield + Hypoxanthine extraction yield + Xanthine extraction yield bExtraction yield (%) = (purine content in sample (mg)/sample mass (kg)) × 100 [file peerj-07-6690-s003.docx]

Table S2. The Box–Behnken experimental design of mix-acid method with three independent variables

| No. | Hydrolysis temperature (℃) | TFA concentration (%) | Hydrolysis time (min) | ^a^Total extraction yield |
| --- | --- | --- | --- | --- |
| 1 | 0 (90) | 0 (85) | 0 (10) | 104.71 |
| 2 | 1 (100) | 0 (85) | 1 (15) | 100.15 |
| 3 | 0 (90) | 0 (85) | 0 (10) | 104.72 |
| 4 | -1 (80) | 0 (85) | 1 (15) | 100.95 |
| 5 | -1 (80) | -1 (80) | 0 (10) | 101.31 |
| 6 | 0 (90) | -1 (80) | -1 (5) | 100.43 |
| 7 | 0 (90) | 0 (85) | 0 (10) | 103.97 |
| 8 | 1 (100) | 1 (90) | 0 (10) | 103.13 |
| 9 | 0 (90) | 1 (90) | -1 (5) | 103.11 |
| 10 | 0 (90) | 1 (90) | 1 (15) | 102.62 |
| 11 | 1 (100) | 0 (85) | -1 (5) | 101.22 |
| 12 | 0 (90) | 0 (85) | 0 (10) | 103.75 |
| 13 | -1 (80) | 0 (85) | -1 (5) | 99.18 |
| 14 | 1 (100) | -1 (80) | 0 (10) | 99.60 |
| 15 | 0 (90) | 0 (85) | 0 (10) | 104.17 |
| 16 | 0 (90) | -1 (80) | 1 (15) | 103.04 |
| 17 | -1 (80) | 1 (90) | 0 (10) | 99.52 |

^a^Total extraction yield (%) = Adenine extraction yield + Guanine extraction yield + Hypoxanthine extraction yield + Xanthine extraction yield

^b^Extraction yield (%) = (purine content in sample (mg)/sample mass (kg)) × 100
